# *Lactococcus lactis* subsp. *cremoris* Produces Zinc Protoporphyrin IX Both Aerobically and Anaerobically and Improves the Bright Red Color of Fermented Meat Products

**DOI:** 10.3390/foods9111583

**Published:** 2020-10-31

**Authors:** Md. Kauser-Ul-Alam, Yu Toba, Shoji Hioki, Toru Hayakawa, Haruto Kumura, Jun-ichi Wakamatsu

**Affiliations:** 1 Laboratory of Applied Food Science, Graduate School of Agriculture, Hokkaido University, Sapporo 060-8589, Japan; kauser71_sust@yahoo.com (M.K.-U.-A.); toruhaya@anim.agr.hokudai.ac.jp (T.H.); kumura@anim.agr.hokudai.ac.jp (H.K.); 2 Field Science Center for Northern Biosphere, Hokkaido University, Sapporo 060-8589, Japan; yu.toba@fsc.hokudai.ac.jp (Y.T.); hioki@fsc.hokudai.ac.jp (S.H.)

**Keywords:** *Lactococcus lactis* subsp. *cremoris*, dry-cured fermented sausage, improvement of color, zinc protoporphyrin IX

## Abstract

This study assessed the color improvement via zinc protoporphyrin IX (ZnPP) formation in nitrite-free, dry-cured sausages processed using five varieties of ZnPP-forming lactic acid bacteria (LAB). The ZnPP contents and color intensity of the sausages and other technological properties were analyzed during the processing of sausages. LAB count and acidity significantly increased in the LAB-inoculated sausages compared to the control group. The bright red color was observed both inside and outside the sausages inoculated with *Lactococcus lactis* subsp. *cremoris* and *Leuconostoc lactis*. However, a brown color was observed on the surface of the sausage inoculated with *Lactobacillus* spp. The redness of *Lactococcus lactis* subsp. *cremoris*-inoculated sausages was close to that of the nitrite-added group. Moreover, the external bright red color was improved by *Lactococcus lactis* subsp. *cremoris* due to the aerobic formation of ZnPP. Therefore, *Lactococcus lactis* subsp. *cremoris* can be used to improve the color of fermented meat products.

## 1. Introduction

Nitrites and nitrates are commonly used coloring agents in meat products with antimicrobial and antioxidant properties [1]. However, under acidic conditions, nitrites are converted to nitrous acid and react with secondary amines, forming N-nitrosamines in the cured meat products [2]. Reports have established that these N-nitrosamines often act as life-threatening carcinogens to humans [3]. Hence, nitrates/nitrites used to cure meat products have been classified as class one agents and proven to be carcinogenic to humans by the International Agency for Research on Cancer [4]. Therefore, finding an effective but harmless alternative to nitrite/nitrate has gained importance in research pertaining to meat-processing. Zinc protoporphyrin IX (ZnPP) is a bright-red-colored metalloporphyrin that is formed by the coordination of divalent zinc into protoporphyrin IX (PPIX) in nitrite/nitrate-free dry-cured ham [5,6] and nitrite-free dry-cured fermented sausages [7]. The bright red color of ZnPP persists irrespective of light or heat exposure [8,9]. Therefore, ZnPP might be a substitute for nitrates/nitrites in improving the color of meat products.

Although ZnPP-forming bacteria are not used in meat products, some bacteria can form ZnPP in salted minced meat. The addition of bacteria such as *Carnobacterium divergens, Serratia liquefaciens* [10], *Lactococcus lactis, Leuconostoc mesenteroides,* and *Enterococcus faecium* [11] lead to the formation of high ZnPP in salted minced meat, thereby improving the color. Interestingly, most ZnPP-forming bacteria are lactic acid bacteria (LAB). Usually, food-grade LAB is used as a bio-preservative against pathogens in meat products and is recognized as safe [12]. However, *Carnobacterium divergens* and *Serratia liquefaciens* are not food-grade bacteria. Moreover, accumulation of biogenic amines by *Leuconostoc mesenteroides* and *Enterococcus faecium* [13] makes their use inappropriate in meat products. Subsequently, five high-food-grade ZnPP-forming LAB were screened: *Enterococcus faecium*, *Lactobacillus curvatus*, *Lactobacillus plantarum*, *Lactococcus lactis* subsp. *cremoris*, and *Leuconostoc lactis* [14]. These LAB have shown the potential to form ZnPP anaerobically in salted minced meat and improved the color. However, such ZnPP-forming LAB have not been practically applied for color improvement in the processing of meat products.

The color of meat products is a critical parameter among consumers for assessing the quality. ZnPP, as a red pigment, is naturally present in meat and is effective in improving the color of meat products. However, the formation of ZnPP is inhibited in the presence of oxygen [15]. Moreover, the distribution of ZnPP in the periphery of the cross-section of parma ham is partially indicated by the weak fluorescence of ZnPP [16]. An important meat-inherent enzyme, ferrochelatase (FECH), is a key contributor to ZnPP formation [17], strictly under anaerobic conditions [18]. It was reported that the tan or brown color of uncured meat is due to the formation of a pigment called metmyoglobin, which is formed due to the oxidation of iron in myoglobin and oxymyoglobin upon continuous exposure to light and oxygen [19]. Thus, the surface of the meat might not show a red coloring due to the inhibition of ZnPP formation by oxygen. ZnPP formation by LAB in salted minced meat has been observed in the absence of oxygen [10,11,14]. LAB are facultative anaerobic organisms and grow well in both the presence and absence of oxygen [20] by switching their metabolism. Hence, there are at least some LAB that are capable of forming ZnPP in the presence of oxygen in meat products. Consequently, aerobic ZnPP formation by LAB might produce a bright red coloration in the nitrite-free meat products.

This study hypothesized that some ZnPP-forming LAB might impart the bright red color to dry-cured meat products by forming ZnPP. Hence, nitrite-free dry-cured sausages were manufactured using the above-mentioned five food-grade ZnPP-forming LAB. The present study was conducted to investigate the effects of ZnPP-forming LAB on the color improvement of dry-cured sausages and to establish the relationship between ZnPP formation and color improvement in meat products. Furthermore, other technological properties regarding dry-cured sausages were also examined to compare their performance levels as desired characteristics.

## 2. Materials and Methods

### 2.1. Preparation of Starter Culture

#### 2.1.1. Starter Culture for Sausage

Previously, five high-ZnPP-forming food-grade LAB, *Enterococcus faecium*, *Lactobacillus curvatus*, *Lactobacillus plantarum*, *Lactococcus lactis* subsp. *cremoris,* and *Leuconostoc lactis*, were isolated from various environmental sources [14] and used as starter cultures in the dry-cured sausages. All LAB were cultivated anaerobically on MRS (De Man, Rogosa and Sharpe) broth (Oxoid Ltd., Basingstoke, Hampshire, UK) at 30 °C. After 24 h of incubation, the concentration of bacteria in the cultured broth was quantified using a hemocytometer under a phase-contrast microscope (Olympus Co., BX50, Tokyo, Japan) to calculate the required amount of bacterial cell suspension per the weight of the meat (6 log colony forming units (CFU)/g of meat). The required bacterial cell suspension was then centrifuged (Tomy Digital Biology Co., CAX-371, Tokyo, Japan) at 10,000× *g* for 15 min at 4 °C to remove the broth (supernatant). The pellets containing crude cells were then washed twice with a sterile physiological saline solution using the same centrifugation technique. Finally, the washed pellets were re-suspended in 10 mL of sterile physiological saline solution, which was used as the starter culture.

#### 2.1.2. Bacterial Culture for Aseptic Meat Homogenate Model System

LAB were cultured in nutrient broth and allowed to grow for 24 h anaerobically at 30 °C. Before using the bacteria in the meat homogenate model experiment, the concentration of bacteria was quantified as previously described in Section 2.1.1, and then adjusted to the appropriate number (6 log CFU/mL of meat homogenate) using sterilized physiological saline.

### 2.2. Preparation of Sausage

Sausages were prepared twice using a mixture of porcine *Longissimus thoracis et lumborum* (LTL) muscle from six different primal cuts of common domestic crossbred pork of loins (Hokkaido, Japan) each time. Sausage manufacturing was carried out in a pilot manufacturing scale plant at the Hokkaido University Agri-Food Center (Hokkaido, Japan). First, the fat and connective tissue were trimmed from the LTL muscles and cut into small blocks. After that, the pieces of the block were chopped, transferring them into a meat grinder with a 3.2 mm plate hole size. Subsequently, salt (2.5%) and glucose (1%) were added to the chopped meat and firmly mixed. Then, the chopped and blended meat was divided into seven groups. Among them, the non-inoculated and nitrite-added groups (300 ppm of final volume) were designated as the control and positive control, respectively. The other five groups were inoculated with five ZnPP-forming LAB (6 log CFU/g of meat), hereby referred to as *Enterococcus faecium* (EB), *Lactobacillus curvatus* (LC), *Lactobacillus plantarum* (LP), *Lactococcus lactis* subsp. *cremoris* (LLC), and *Leuconostoc lactis* (LL). Sterile ultra-pure water was added to the non-inoculated group instead of the bacterial solution. Then, the mixture of each group was stuffed into a cellophane casing (Tohcello bista SP-S 450, Shikoku Tohcello Co. Ltd., Tokushima, Japan) with a stuffer machine. Five sausages (approximately 150 g in weight and 12 cm in length) from each group were prepared and placed into an incubation chamber. A thermal hygrostat (QBX–132 HRST 1, Fukushima Galilei Co. Ltd. Osaka, Japan) was used as the incubation chamber. The sausages of each group were subjected to the following conditions: resting period at 1 °C for 24 h, fermentation period at 18 °C with 85% relative humidity (RH) for the next 7 days, drying period at 14 °C with 75% RH for the next 7 days, and ripening period at 12 °C with 80% RH for 14 days. Seven sausages (one from each group) were chosen at random and analyzed on every seventh day of processing. For each sausage, three replications have been performed.

### 2.3. The Total and Lactic Acid Bacterial Counts

To determine the bacterial counts, 3 g of the sample was obtained aseptically from the core portion of the sausages and transferred to a sterile plastic bag. Then, the sample was homogenized with 27 mL of sterile saline (0.9% salt) using a stomacher (Exnizer 400, Organo, Tokyo, Japan). Ten-fold serial dilutions were performed, and suitable dilutions (0.1 mL) were poured into the Petri dish in duplicate. The Petri dishes containing two different growth media, standard plate count (SPC) agar (Eiken, Chemical Co. Ltd., Tochigai, Japan) for the total bacterial count and MRS agar (Oxoid, Kanto Chemical Co. Ltd., Tokyo, Japan) for the total lactic acid bacterial count, were used. The dilutions were spread onto the plate using a sterilized glass rod. Subsequently, the SPC agar plate was incubated aerobically at 30 °C for 24 h, and the MRS agar plate was incubated anaerobically at 30 °C for 48 h. Finally, the number of bacteria was calculated and expressed as log CFU/g.

### 2.4. Water Activity, pH, and Titratable Acidity

The water activity of the sausages was determined using a LabMaster meter (Novasina AG, Lachen, Switzerland) at 25 °C. The pH of the sausages was measured with a pH meter (Horiba, F-55 series, Horiba Ltd., Kyoto, Japan) by dipping the probe into a 20% meat homogenate. The titratable acidity of the sausages was estimated by titration of the sample with 0.1 N NaOH using phenolphthalein (1% alcoholic phenolphthalein) as an indicator [21].

### 2.5. Imaging and ZnPP Autofluorescence

For visual imaging and to observe ZnPP autofluorescence (ZAF) in the dry-cured sausages, a digital camera (Nikon, D3300, Tokyo, Japan) was used as mentioned previously [10] For ZAF, two sheet-type band-pass filters were used. The filters transmit about 600 nm light (equipped with a digital camera) (BPB-60, Fujifilm Co., Tokyo, Japan) and approximately 420 nm light (provided with purple LED (Light-emitting diode) lighting devices) (BPB-42, Fujifilm Co., Tokyo, Japan), respectively.

### 2.6. Measurement of ZnPP in Dry-Cured Sausages

Extraction of ZnPP using cold acetone and analysis of fluorescence were carried out as previously described with minor modifications [22]. First, the chopped samples obtained from the sausages were soaked in water for 1 h at room temperature for softening, and 20% homogenate of the sample was prepared. Next, 1.5 mL of the meat homogenate was obtained, and 75% cold acetone was added to it as the final volume to extract ZnPP. The sample was kept at 4 °C for 30 min in the dark. Finally, after filtering the extract through a filter paper (No. 2, 90 mm, Toyo Roshi Kaisha Ltd., Tokyo, Japan), the fluorescence intensity of ZnPP in the extract was measured using a spectrofluorophotometer (RF-5300PC; Shimadzu Co., Kyoto, Japan) at Ex/Em: 420/590 nm. Thus, the fluorescence intensity was considered as the amount of ZnPP formed and expressed as nmol/g DM (dry matter). ZnPP (Aldrich, Chem. Co., Milwaukee, WI, USA) was used as the standard.

### 2.7. Color Analysis

Color measurements were conducted with a spectrocolorimeter (CM-700d, Konica Minolta, Tokyo, Japan) using an 8 mm port size, illuminant D65, and 10° standard observer. The colorimeter was first calibrated using a white standard plate. For measuring the color on the surface of sausages, the outer portion of the sausages was cut up to a depth of 2 mm to obtain a uniform surface. For measurement of the color inside the sausages, the sausages were cut into 2.5 cm thick cross-sections to obtain a uniform shape. Each cross-section was then exposed to air for approximately 1 h at room temperature to bloom (allowing the remaining heme pigments to oxygenate). Finally, the color was measured carefully, applying gentle pressure to ensure that no light entered or exited the aperture and carefully avoiding any fat particles. The values were considered as L* (lightness), a* (redness), and b* (yellowness), and were obtained from three different cross-sections of each group. The hue angle and chroma were also calculated.

### 2.8. Aseptic Meat Homogenate Model System and Measurement of ZnPP Fluorescence Intensity

To investigate the ZnPP-forming ability of the inoculated LAB under aerobic conditions, an aseptic meat homogenate model experiment was carried out as previously described [10], with minor modifications. First, 30% pork homogenate was aseptically prepared from the core of the LTL muscle using a sterilized cup and homogenizer (CELL MASTER CM-100, AZ ONE Co. Tokyo, Japan) at 10,000 rpm for 1.5 min. Then, 0.9 mL of 30% pork homogenate, 0.45 mL of 10% salt solution, and 0.15 mL of broth containing specific LAB isolates were transferred to sterilized test tubes (final concentrations: 20% pork homogenate, 3% salt, and 2.0 × 10^6^ CFU/mL). To confirm the sterility of the preparation, antibiotics were added to the antibiotic-treated group at a final concentration of 70 µg/mL penicillin G potassium, 250 µg/mL streptomycin sulfate, and 50 µg/mL gentamicin sulfate. The different samples (model mixtures) were then incubated aerobically and anaerobically at 25 °C for 7 days in the dark. An oxygen-impermeable storage bag containing oxygen absorbent (A-500HS, I.S.O. Inc., Yokohama, Japan) was used to regulate the anaerobic condition. Finally, the fluorescence intensity of ZnPP in the incubated sample was measured according to Section 2.6.

### 2.9. Statistical Analysis

Data were expressed as mean ± standard error. One-way analyses of variance (ANOVA) with Tukey’s multiple comparison tests were performed to evaluate the differences among individuals. The values were calculated using Microsoft Excel 2013 (Microsoft Corp., Redmond, WA, USA) with Ekuseru–Toukei 2012 (Social Survey Research Information, Tokyo, Japan) as add-in software. The value of *p* < 0.05 was considered statistically significant.

## 3. Results

### 3.1. Changes in Technological Properties of Dry-Cured Sausages

Initially, the technological properties of the sausages were inspected in terms of changes in weight and water activity (a_w_), microbial profiles, pH, and titratable acidity as a percentage of lactic acid. The properties of the sausages were analyzed every seven days for 28 days. The first and second weeks of processing were considered as periods of “fermentation (0–7 days)” and “drying (7–14 days)”, respectively, whereas the last two weeks were considered as a period of “ripening (14–28 days)” for the sausages.

**Figure 1:**
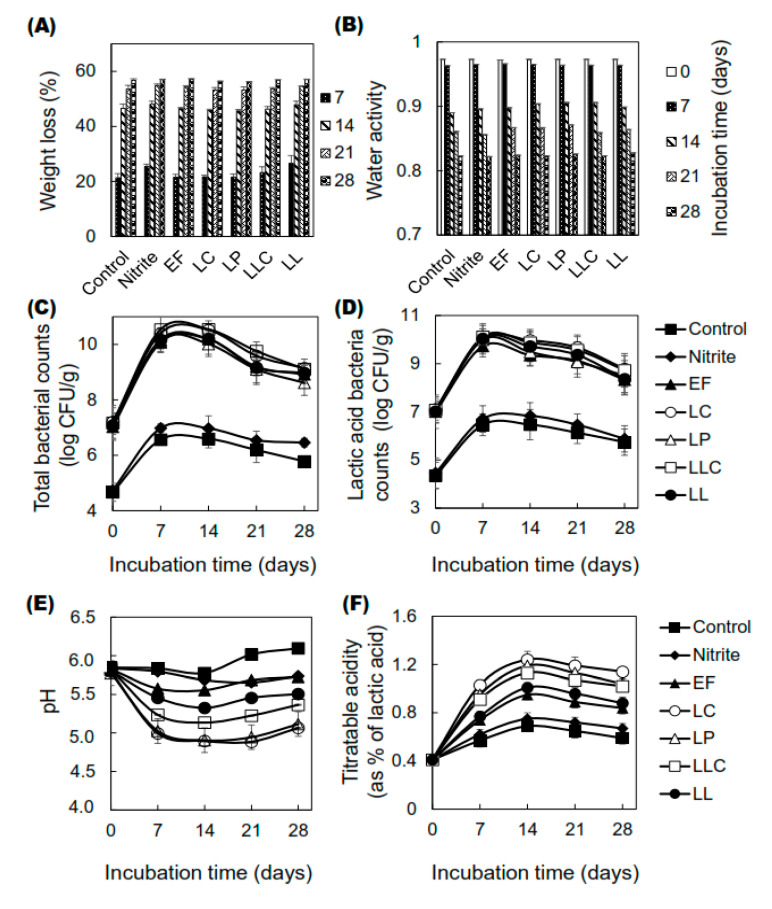
Changes in the technological properties of dry-cured fermented sausages during processing. Five high zinc protoporphyrin IX-(ZnPP)-forming lactic acid bacteria (LAB) as starter cultures (6 log colony forming units (CFU)/g) were used to prepare dry-cured sausages. After every 7 days of processing, the sausages were analyzed for 28 days. Changes in weight loss, (**A**) and water activity, (**B**) the evolution of total bacterial counts, (**C**) and lactic acid bacterial counts (**D**), and changes in pH (**E**) and titratable acidity as % of lactic acid (**F**) of dry-cured fermented sausages during processing are indicated. Bars represent standard errors of the mean (*n* = 2). ZnPP: zinc protoporphyrin IX, LAB: lactic acid bacteria, CFU: colony forming units, EF: *Enterococcus faecium*, LC: *Lactobacillus curvatus*, LP: *Lactobacillus plantarum*, LLC: *Lactococcus lactis* subsp. *cremoris*, LL: *Leuconostoc lactis.*

Regarding weight loss and water activity, there was no significant difference (*p* > 0.05) observed among the groups of sausages inoculated with ZnPP-forming LAB and non-inoculated ones. The weight of each sausage was reduced noticeably during the fermentation and drying periods and then decreased gradually during the ripening stage (Figure 1A). The weights of sausages substantially reduced by 45% to 48% over the first 14 days of processing and 56% to 57% by the end of ripening compared to the initial weight. The water activity of the treated sausages decreased steadily (Figure 1B) throughout the process. Initially, the water activity values of all groups were approximately 0.972–0.973. By the end of ripening, the water activity was reduced to 0.822–0.827.

Subsequently, the microbiological profiles (total bacterial count and lactic acid bacterial count) were analyzed to determine the viability of the bacteria in the sausages (Figure 1C,D). Significant differences (*p* < 0.05) were observed in the inoculated sausages compared to the control and nitrite-added groups in terms of the total bacterial count (Figure 1C) and lactic acid bacterial count (Figure 1D). The maximum value for the total bacterial count was noticed by day seven of the fermentation period, and a slight decrease was observed at the end of ripening. As for the total bacterial count at the end of fermentation, the highest levels were observed in the LLC group (10.55 log CFU/g) followed by the LC group (10.52 log CFU/g) and showed significant differences (*p* < 0.05) compared to the control and nitrite-added group. In contrast, the control and nitrite-added groups showed values of 6.60 and 6.97 log CFU/g, respectively. At the end of the ripening period, the highest count of 9.12 log CFU/g was observed for the LLC group, and the lowest count of 5.78 log CFU/g was observed for the control group. At the end of the fermentation period, the maximum value for the total LAB count (Figure 1D) was observed in the LLC group (10.11 log CFU/g), whereas the minimum value was observed in the control group (6.46 log CFU/g). After fermentation, the LAB counts in all the groups slightly decreased. At the end of the ripening period, the counts were approximately 8.5 log CFU/g in all the LAB-inoculated groups and 5.8 log CFU/g in the control and nitrite-added groups.

The pH and acidity were used as parameters to analyze the amount of lactic acid formed in the sausages. After seven days of fermentation, the pH significantly decreased from the initial value (~5.80) to 5.56, 5.00, 5.03, 5.24, and 5.46 in the EF, LC, LP, LLC, and LL groups, respectively (*p* < 0.05) (Figure 1E). However, the pH changed non-significantly in the control and nitrite-added groups. The lowest pH was observed in the LP and LC groups (4.90), followed by the LLC group (5.14) after 14 days of drying. After 21 days, the pH progressively increased until the end of ripening in each sausage. Lactic acid accumulated more rapidly during the fermentation and drying periods and then slightly decreased (Figure 1F). At day seven, the acidity significantly increased from the initial value (~0.41%) to 0.74, 1.03, 0.95, 0.91, and 0.77% in the EF, LC, LP, LLC, and LL groups, respectively (*p* < 0.05). In contrast, there was no significant change in the control and nitrite-added groups. The maximum amount of lactic acid was measured in the LP (1.24%) and LC (1.19%) groups, followed by the LLC group (1.13%) after 14 days of drying.

### 3.2. Evaluation of the Color-Improving Ability of High ZnPP-Forming LAB in Dry-Cured Sausages

#### 3.2.1. Changes in the External Color of Dry-Cured Sausages

**Figure 2:**
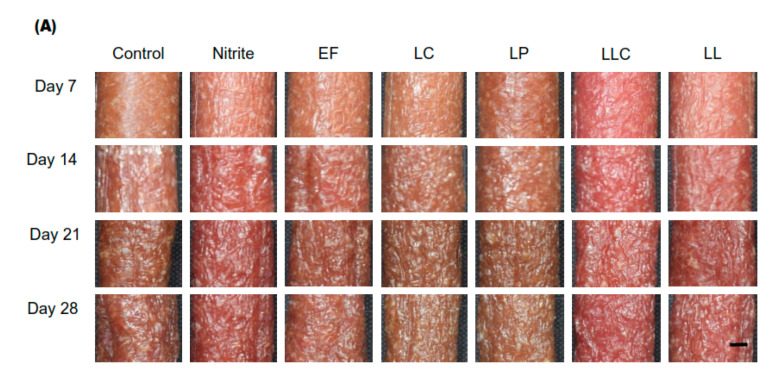

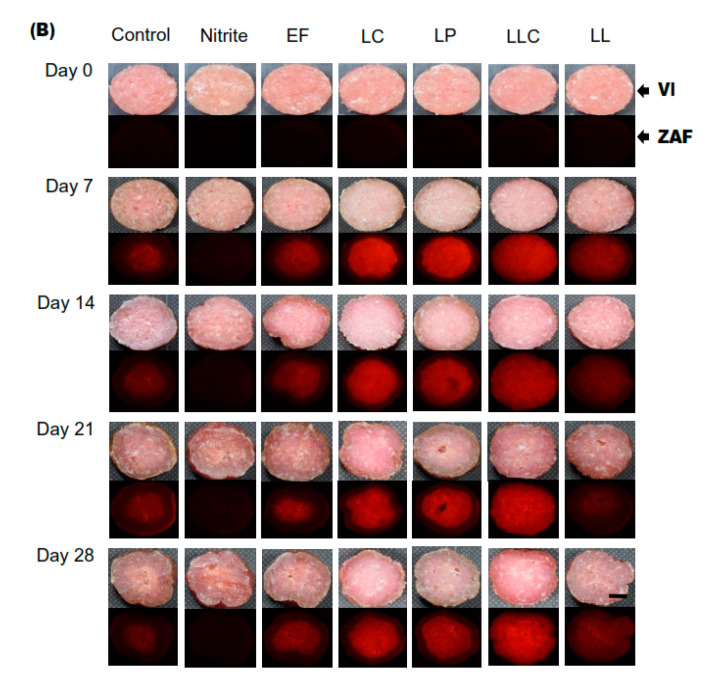
Effects of high ZnPP-forming LAB on the color of dry-cured fermented sausages. Dry-cured sausages inoculated with high ZnPP-forming LAB were ripened up to 28 days. Change in the color of the surface (**A**) and the internal color and ZnPP distribution (**B**) during processing. The upper ones are visual images (Vis) and the lower ones are ZnPP autofluorescence images (ZAFs), as indicated by arrows. Scale bars: 1 cm. ZnPP: zinc protoporphyrin IX, LAB: lactic acid bacteria, Vis: visual images, ZAFs: ZnPP autofluorescence images, EF: *Enterococcus faecium*, LC: *Lactobacillus curvatus*, LP: *Lactobacillus plantarum*, LLC: *Lactococcus lactis* subsp. *cremoris*, LL: *Leuconostoc lactis.*

To investigate the effects of ZnPP-forming LAB on the external color, visual images were observed throughout the process (Figure 2A). An increase in the bright red color was observed in the nitrite-added group after 14 days of drying, and this color stabilized by the end of ripening. Among the LAB-inoculated groups, the sausages of the LLC group showed a bright red color after seven days of fermentation and retained the redness up to the end of ripening compared to the non-inoculated group. The color was similar to that of the nitrite-added group and more intensely red than the sausages of the LL and EF groups. In contrast, the brown color on the surface of the sausages was observed in the LC and LP groups after seven days of fermentation and gradually increased up to the end of ripening compared to that in the non-inoculated and other inoculated groups. Thus, two distinct colors were obtained for the external surface of sausages depending on the ZnPP-forming LAB.

#### 3.2.2. Changes in the Internal Color of Dry-Cured Sausages and Observation of ZnPP Distribution

To investigate the effects of ZnPP-forming LAB on the internal color, visual and ZAF images were observed in the cross-section of sausages throughout the process (Figure 2B). The intensity of the bright red color gradually increased in the LLC and LC groups from seven days followed by the LP, LL, and EF groups, and the color was retained for 28 days. The color was similar to that observed for the nitrite-added group. Sausages in the LLC group also showed the strongest fluorescence, followed by those in the LC, LP, LL, and EF groups compared with that in the non-inoculated group. Interestingly, a weakly fluorescent ring of ZnPP was observed along the circumference of the cross-section of the sausages, especially in the LC and LP groups. In contrast, no fluorescent ring of ZnPP was observed outside the sausages inoculated with *Lactococcus lactis* subsp. *cremoris* (LLC group). Additionally, almost no, or very weak, ZAF was observed in the nitrite-added and control groups throughout the process.

#### 3.2.3. Changes in the ZnPP Content of Dry-Cured Sausages

To verify the ZnPP-forming ability of LAB in dry-cured sausages, the amount of ZnPP was measured (Table 1). On day seven, the amount of ZnPP in the LAB-inoculated sausages dramatically increased and was significantly higher (*p* < 0.01) than that in the control and nitrite-added groups. The highest content of ZnPP was found in the LLC group, which showed non-significant difference compared with the LC group after seven days of fermentation. Moreover, from day seven onwards, the ZnPP content in the LAB-inoculated sausages steadily increased and was also higher than that in the control and nitrite-added groups (*p* < 0.01). At the end of ripening, the highest contents of ZnPP were observed in the LC and LLC groups, respectively. Although an increase in ZnPP with respect to time was measured to some extent in the control group, the amount of ZnPP in the nitrite-added group was negligible throughout the process.

#### 3.2.4. Color Profiles of the Dry-Cured Sausages

**Figure 3:**
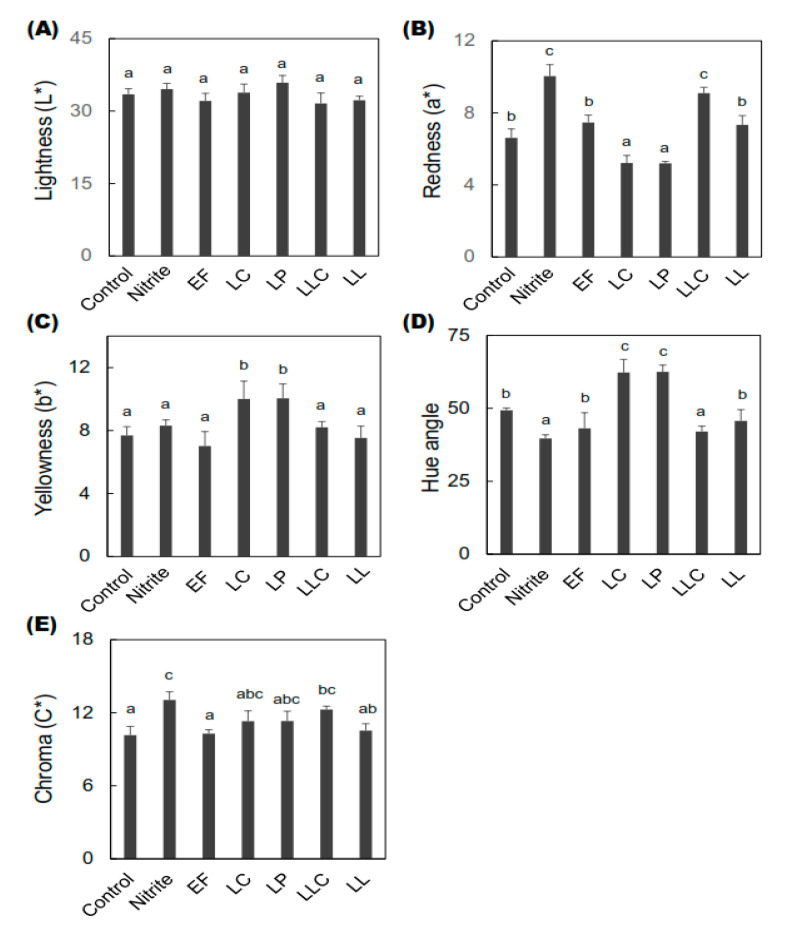
Effects of high ZnPP-forming LAB on the external color profiles of dry-cured fermented sausages. CIE (Commission internationale de l’éclairage) L* (lightness), a* (redness), b* (yellowness), hue angle, and chroma values of the fermented sausages were measured after 28 days of ripening. The figures indicate (**A**) lightness (L*), (**B**) redness (a*), (**C**) yellowness (b*), (**D**) hue angle, and (**E**) chroma (C*) values of the sausages at the end of ripening. Bars represent standard errors (*n* = 2). a–c: columns with different letters in the different groups differ significantly (*p* < 0.05). ZnPP: zinc protoporphyrin IX, LAB: lactic acid bacteria, EF: *Enterococcus faecium*, LC: *Lactobacillus curvatus*, LP: *Lactobacillus plantarum*, LLC: *Lactococcus lactis* subsp. *cremoris*, LL: *Leuconostoc lactis.*

**Figure 4:**
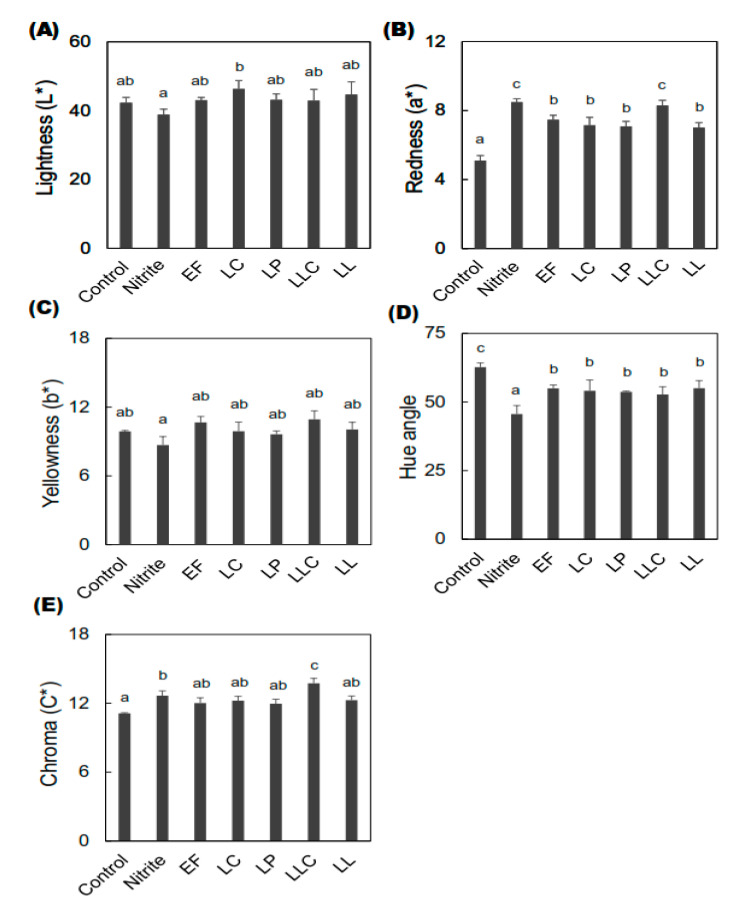
Effects of high ZnPP-forming LAB on the internal color profiles of dry-cured fermented sausages. To measure the color of the inner side of the sausages after 28 days of ripening, the sausages were cut into 2.5 cm thickness with uniform shape and exposed to air for about 1 h to bloom. The figures indicate (**A**) lightness (L*), (**B**) redness (a*), (**C**) yellowness (b*), (**D**) hue angle, and (**E**) chroma (C*) values of the sausages at the end of ripening. Bars represent standard errors of the means (*n* = 2). a–c: columns with different letters in the different groups differ significantly (*p* < 0.05). ZnPP: zinc protoporphyrin IX, LAB: lactic acid bacteria, EF: *Enterococcus faecium*, LC: *Lactobacillus curvatus*, LP: *Lactobacillus plantarum*, LLC: *Lactococcus lactis* subsp. *cremoris*, LL: *Leuconostoc lactis.*

The color profiles of both external and internal dry-cured sausages were analyzed using the CIE (Commission internationale de l’éclairage) L* (lightness), a* (redness), and b* (yellowness) colorimetric system after 28 days of ripening (Figure 3 and Figure 4).

In terms of external color, no significant difference in the L* values was observed in the groups. However, the highest and lowest L* values among the groups were found in the LP and LLC groups, respectively (Figure 3A). Moreover, among the LAB-inoculated groups, the LLC group showed a significantly higher a* value than that in the control group, and this result was similar to that of the nitrite-added group. The LC and LP groups showed significantly lower a* values than that in the control group (Figure 3B). Moreover, the b* values of the LC and LP groups were significantly higher than those of the other groups (Figure 3C). Furthermore, the LLC group showed a significantly lower hue angle value compared to the control group, and this result was similar to that of the nitrite-added group (Figure 3D). In contrast, the LC and LP groups showed a significantly higher hue angle than the control group. However, the highest chroma (C*) value among the LAB-inoculated sausages was also found in the LLC group (Figure 3E). The C* values in the LLC group were significantly higher compared with that in the control group (Figure 3E), and this result was similar to that of the nitrite-added group.

In terms of internal color (the core part of the sausages), the L* value of the nitrite-added group was significantly lower than that of the LC group (Figure 4A). All the inoculated groups showed significantly higher a* values than the control group (Figure 4B). The highest a* value among the inoculated groups was observed in the LLC group, similar to the nitrite-added group. No significant difference in the b* value was observed for the inoculated sausages compared to the control and nitrite-added groups (Figure 4C). Moreover, a significantly lower hue angle was observed in all the LAB-inoculated sausages than that in the control group (Figure 4D). However, the highest C* value (Figure 4E) was observed in the LLC group, even more than that of the nitrite-added group.

### 3.3. Effect of Oxygen on ZnPP Formation by the ZnPP-Forming Inoculated-LAB

**Figure 5:**
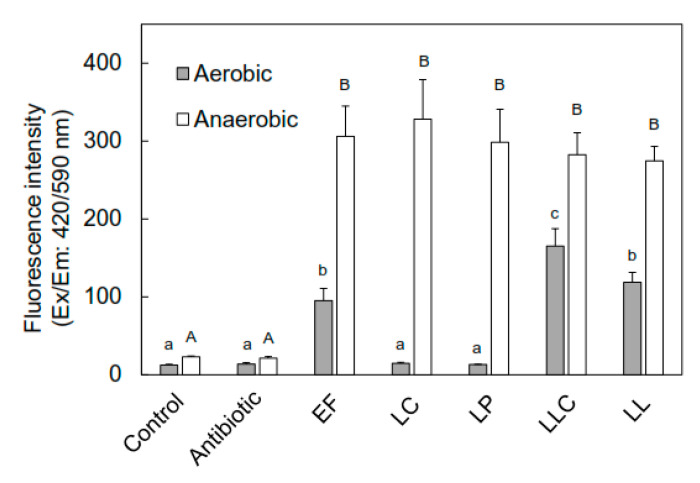
Effects of oxygen on ZnPP formation by the ZnPP-forming LAB in porcine *Longissimus thoracis et lumborum* (LTL) muscle homogenate. A mixture containing the final concentrations of 20% pork homogenate, 3% salt, and 2.0 × 10^6^ CFU/mL of respective LAB was incubated both aerobically and anaerobically at 25 °C for 7 days in the dark. After incubation, ZnPP of the mixture was extracted using the 75% acetone method, and the fluorescence intensity (Ex/Em: 420/590 nm) was measured. Bars represent the standard error of the means (*n* = 3). a–c and A–B: columns with different letters in the different groups differ significantly (*p* < 0.01). ZnPP: zinc protoporphyrin IX, LAB: lactic acid bacteria, EF: *Enterococcus faecium*, LC: *Lactobacillus curvatus*, LP: *Lactobacillus plantarum*, LLC: *Lactococcus lactis* subsp. *cremoris*, LL: *Leuconostoc lactis.*

To investigate the effect of oxygen on LAB-associated ZnPP formation, an aseptic homogenate meat model experiment was performed, and the LAB-inoculated homogenate was incubated both aerobically and anaerobically. After seven days of incubation, the highest fluorescence intensity of ZnPP was observed in the presence of *Lactococcus lactis* subsp. *cremoris* and was significantly different from that observed in the presence of the other LAB, *Leuconostoc lactis,* and *Enterococcus faecium*, under aerobic conditions (Figure 5). They also formed ZnPP under anaerobic conditions. In contrast, no fluorescence was observed in the presence of *Lactobacillus curvatus* and *Lactobacillus plantarum* under aerobic conditions, although the highest fluorescence intensity of ZnPP was observed in the presence of *Lactobacillus curvatus* under anaerobic conditions compared to that observed for the control and other LAB-inoculated groups (Figure 5).

## 4. Discussion

In this study, high-ZnPP-forming LAB were used as starter cultures in dry-cured fermented sausages. The bright red color of the sausages increased during processing. The technological attributes of the dry-cured sausages were also examined throughout the manufacturing process.

### 4.1. Internal Color and Anaerobic ZnPP Formation

The ZAF and bright red color observed in all the ZnPP-forming LAB-inoculated, dry-cured sausages from day seven to the end of ripening were due to the formation of ZnPP by the ZnPP-forming LAB. It is thought that LAB growth causes acidic fermentation in meat products, and the end products of this process, mainly organic acids, accumulate in the extracellular environment and reduce the pH of the medium [23]. pH is considered a crucial factor for the formation of ZnPP in meat and meat products, and the formation of ZnPP declines greatly at very low or high pH [21]. The optimum pH for the formation of ZnPP is approximately 5.5–6.0 [24]. In this study, lowering of the pH was observed in LAB-inoculated sausages after seven days of fermentation, and the formation of ZnPP and ZAF was consistent with the lowering of pH. Thus, the inoculated ZnPP-forming LAB might make the medium of meat products suitable for ZnPP formation by reducing the pH during bacterial fermentation. It is also assumed that ZnPP formation in the LAB-inoculated groups might be due to the proteolytic breakdown of myoglobin [25]. Although endogenous enzymes present in dry-fermented sausages are primarily responsible for protein degradation, bacterial proteases and peptidases contribute to the initial breakdown of myofibrillar and sarcoplasmic proteins (e.g., hemoglobin/myoglobin) [26]. Therefore, most of the inoculated LAB made the sausages suitable for ZnPP formation by reducing the pH and proteolytic action, thereby improving the internal bright red color.

The ring formation on the outer surface of the sausage cross-section (Figure 2B) was due to the absence of ZnPP, *Lactobacillus* spp. cannot form ZnPP in the presence of oxygen. In LAB-inoculated sausages, the FECH enzyme and other metabolites derived from the ZnPP-forming LAB might contribute to ZnPP formation. It was claimed that the FECH of yeast and bacteria could convert myoglobin-heme and heme from meat to ZnPP via the replacement of iron in the protoporphyrin ring by zinc ions [18]. Moreover, almost no ZAF and very weak fluorescence were observed in the nitrite-added group due to nitric oxide derived from nitrite/nitrate that inhibited the formation of ZnPP during processing [10]. On the other hand, very weak ZAF and fluorescence have been observed in the control group due to the meat-inherent mechanisms of ZnPP formation [17] or bacteria in the raw meat or bacterial contamination during processing of the sausages. Therefore, regarding the internal color of the dry-cured sausages, all ZnPP-forming LAB used in this study improved the color by forming ZnPP anaerobically.

### 4.2. External Color and Aerobic ZnPP Formation

The sausages inoculated with mainly *Lactococcus lactis* subsp. *cremoris* and *Lactobacillus* spp. showed brighter red and brown coloration, respectively, on the external surface. The external surface of sausages inoculated with *Lactococcus lactis* subsp. *cremoris* and *Lactobacillus* spp. showed brighter and duller red coloration, respectively. Thus, the higher external a* values of the sausages were consistent with the development of the bright red color. The external bright red coloration of the sausages inoculated with *Lactococcus lactis* subsp. *cremoris* was thought to be due to the formation of ZnPP. This assumption is strengthened by the fact that *Lactococcus lactis* subsp. *cremoris* can form ZnPP in the presence of oxygen. This external redness might be a result of LAB-specific components produced during their metabolism, and a particular component derived from the *Lactococcus lactis* subsp. *cremoris* can contribute to the formation of ZnPP in the presence of oxygen, distinguishing *Lactococcus lactis* subsp. *cremoris* from the other LAB. The bright red color was observed in the nitrite-added group due to the formation of nitrosyl myoglobin [27]. Our findings reveal that the external brown color in the LAB-inoculated sausages increased due to a lack of ZnPP content, indicating the inability of LAB to form ZnPP in the presence of oxygen in dry-cured sausages. The formation of ZnPP on the meat surface that generally occurs due to meat-inherent functions was inhibited due to the presence of oxygen [15]. Chau et al. reported that meat-inherent FECH cannot facilitate the formation of ZnPP in meat via an iron-removal reaction of heme in the presence of oxygen. In addition, the external brown color observed in the control group in this study is thought to have been produced by the oxidation of the major color pigment of fresh meat, namely myoglobin [28]. However, ZnPP-forming LAB, mainly *Lactococcus lactis* subsp. *cremoris* and *Leuconostoc lactis* to a lesser extent, form ZnPP aerobically on the surface of sausages. Therefore, the ZnPP-forming *Lactococcus lactis* subsp. *cremoris* used in this study would improve the external bright red color of dry-cured sausages by forming ZnPP aerobically.

### 4.3. Technological Properties of the Dry-Cured Sausages and ZnPP Formation

In this study, the processing conditions applied in the production of dry-cured sausages were slightly different from the common manufacturing practices in order to facilitate the formation of ZnPP. The fermentation of dry-cured sausages was carried out for a long period of time to achieve strong acidification by the production of lactic acid. Hence, the pH of the inoculated sausages decreased, and the lactic acid content increased during the manufacturing process. The increase in lactic acid content is consistent with the decrease in pH. A decrease in pH in most of the LAB-inoculated sausages indicated that the strains used have intense acidifying activity in the incubating conditions. The pH decrease was caused by an accumulation of organic acids, mainly lactic acid, due to the breakdown of carbohydrates during fermentation [29]. In the inoculated dry-cured sausages, the number of LAB and the lactic acid content increased, since the production rate of lactic acid in the sausages depended on the increase in the number of inoculated LAB. Usually, LAB produce lactic acid by fermenting sugars via glycolysis and the action of lactate dehydrogenase [30]. The growth of LAB is greatly influenced by the fermentation time and temperature during processing. However, the formation of ZnPP is greatly governed by the pH of the medium, and there are two independent mechanisms of ZnPP formation in the porcine skeletal muscles with optimal pH values of 5.5 and 4.75 [31]. Hence, the reduction in pH is important for ZnPP formation because the pH in fresh meats varies from 5.5–6.0 [9,32]. The enzymatic activity in the biological process is also influenced by the pH. The activity of the FECH, a key enzyme for ZnPP formation, exerts different functions at different pH [33], thereby altering ZnPP formation. It is thought that the decrease in pH during dry-cured sausage processing may promote the breakdown of myoglobin/hemoglobin and the formation of PPIX via heme, resulting in the formation of ZnPP. Moreover, the free zinc content is relatively low at the average pH of meat [21]. It is also assumed that at low pH, zinc was released by the degradation of zinc-binding proteins and inserted into the PPIX to form ZnPP. However, fermentation time and temperature are important parameters in the production of dry-cured sausages to improve the color by LAB-forming ZnPP. Therefore, fermentation time and temperature play an important role in the formation of ZnPP in dry-cured fermented sausage by facilitating bacterial growth and subsequent production of organic acids and reduction of pH.

## 5. Conclusions

Thus, ZnPP formation by LAB was demonstrated to be useful for improving the color of nitrite-free meat products. All five bacteria species used in this study improved the internal color of sausages, but a dull external appearance was observed in four LAB-inoculated sausages, except the sausages inoculated with *Lactococcus lactis* subsp. *cremoris*. Only *Lactococcus lactis* subsp. *cremoris* improved the external bright red color of the sausages, and subsequently, the entire ZnPP autofluorescence was observed in the sausages since *Lactococcus lactis* subsp. *cremoris* can form ZnPP both aerobically and anaerobically. Therefore, it was suggested that the high ZnPP-forming *Lactococcus lactis* subsp. *cremoris* could be used as an alternative to nitrites/nitrates for improving the color of meat products.

## Figures and Tables

**Table 1:** Changes in the zinc protoporphyrin IX (ZnPP) content (nmol/g dry matter (DM)) during the production of nitrite-free dry-cured fermented sausages.

	Day 0	Day 7	Day 14	Day 21	Day 28
Control	0.19 ± 0.00 ^a^	1.07 ± 0.01 ^b^	1.41 ± 0.01 ^b^	1.72 ± 0.04 ^b^	2.03 ± 0.03 ^b^
Nitrite	0.15 ± 0.00 ^a^	0.37 ± 0.01 ^a^	0.48 ± 0.02 ^a^	0.54 ± 0.01 ^a^	0.57 ± 0.01 ^a^
EF	0.19 ± 0.00 ^a^	2.04 ± 0.30 ^c^	2.92 ± 0.05 ^c^	3.58 ± 0.05 ^c^	3.80 ± 0.08 ^c^
LC	0.17 ± 0.01 ^a^	3.75 ± 0.04 ^e,f^	4.66 ± 0.05 ^e,f^	5.42 ± 0.12 ^e^	6.10 ± 0.16 ^d^
LP	0.17 ± 0.01 ^a^	3.58 ± 0.03 ^e^	4.37 ± 0.12 ^e^	4.94 ± 0.11 ^e^	5.66 ± 0.08 ^d^
LLC	0.16 ± 0.00 ^a^	3.89 ± 0.02 ^f^	4.87 ± 0.09 ^f^	5.40 ± 0.13 ^e^	6.08 ± 0.12 ^d^
LL	0.19 ± 0.01 ^a^	2.71 ± 0.05 ^d^	3.55 ± 0.06 ^d^	4.17 ± 0.12 ^d^	4.47 ± 0.18 ^c^

Data are expressed as mean ± SEM (*n* = 2). Different letters in the same column indicate significant differences (*p* < 0.01) among the groups.
